# Exploring the role of maternal routine and problem-solving actions in promoting child health and nutrition in Kenyan drylands: a qualitative study

**DOI:** 10.1186/s41043-026-01251-8

**Published:** 2026-02-01

**Authors:** Patricia Jebet Kiprono, Oliver Hensel, Brigitte Kaufmann

**Affiliations:** 1 German Institute for Tropical and Subtropical Agriculture (DITSL), Steinstr. 19, 37213 Witzenhausen, Germany; 2 Agricultural and Biosystems Engineering, Faculty of Organic Agricultural Sciences, University of Kassel, Nordbahnhofstr. 1a, 37213 Witzenhausen, Germany; 3 Social Ecology of Tropical and Subtropical Land‑Use Systems, Institute of Agricultural Sciences in the Tropics (Hans-Ruthenberg-Institute), University of Hohenheim, 70599 Stuttgart, Germany

**Keywords:** Caregivers’ knowledge, Challenges, Practices, Contextual solutions, Infant, Mother, Interventions, Arid and Semi-Arid lands (ASALs), Kenya, Africa

## Abstract

**Background:**

In the drylands of northern Kenya, mothers strive to promote the health and nutritional wellbeing of their children, but face many challenges. Most studies, especially those focusing on (agro-)pastoralists, use a problem-lens, with recommended standard interventions to improve child nutrition that do not necessarily fit the local conditions. This study aims to explore (agro-)pastoral caregivers’ knowledge and their practical solutions in child nutrition and care, uncovering their routine and problem-solving actions.

**Methods:**

The Activity Knowledge Analysis tool maps caregivers’ practices aimed at achieving their goals (routine actions), and identifies the challenges they face, underlying causes, and the problem-solving actions. We used this participatory tool in 18 Focus Group Discussion sessions with caregivers from Rendille, Burji and Borana communities in Marsabit County, Kenya. The discussions were recorded, transcribed and analysed using MAXQDA software.

**Results:**

Mothers explained their routine actions which included age-appropriate feeding practices, maintaining hygiene, and facilitating the child’s developmental milestones to achieve their goal of having a healthy child. Some of the routine actions include providing special diets, responsive feeding; personal, food and environmental hygiene; massage, engagement in play and interaction to facilitate development. The challenges that mothers face include maintaining their own health, difficulties with feeding and delayed developmental milestones. These challenges are further compounded by contextual factors; poverty, time constraints and lack of support. To overcome the challenges, mothers used a range of problem-solving actions, including enriching their children’s diets, diversifying their income sources, borrowing food or money, and seeking social support from family members and the community.

**Conclusion:**

Participatory approaches, such as the use of the Activity Knowledge Analysis tool, have proved useful in exploring caregivers’ knowledge, highlighting their problem-solving actions. These insights can be used to contextualize existing government and stakeholder interventions by incorporating community-led solutions and leveraging local structures for knowledge exchange and dissemination. This approach promotes the use of locally available resources and ensures cultural appropriateness, enhancing the adoption of practices to improve children’s health and nutritional status.

## Background

Child mortality has seen a significant reduction globally over the past two decades, resulting in a decrease in the number of deaths per 1000 live births to 39 children before the age of five years in 2021. However, this reduction is not uniform across all regions, as Sub-Saharan Africa still records a high number of under 5 child mortalities, with 74 deaths per 1000 live births [1]. About 50% of under-five deaths are caused by undernutrition, particularly in sub-Saharan Africa [2].

While some progress has been made in addressing child malnutrition in Kenya, with an average decline in stunting from 40% to 18%, wasting from 7% to 5%, and overweight from 6% to 3% between 1993 and 2022, on average 10% of children under the age of five years remain underweight by 2022 [3]. Malnutrition rates are even higher in the drylands of northern Kenya, such as in Marsabit County. The most recent Standardised Monitoring and Assessment of Relief and Transitions (SMART) survey revealed that 28.3% of the children were stunted, 12.9% were wasted, and 25.5% were underweight [4]. Acute malnutrition in drylands can be attributed to several causes, including immediate causes such as inadequate dietary intake and disease; underlying causes such as household food insecurity, inadequate social and care environment, insufficient health services, and an unhealthy environment; and basic or systemic causes such as environmental conditions, e.g. seasonality and functioning of formal and informal institutions [5].

These causes are experienced at various levels, including child, mother, family/household, and community/society [6]. They not only encompass physiological factors, such as diet and disease [7], but also social factors, such as family support, instrumental factors, such as livelihoods [8], and psychological factors, such as women’s agency and self-confidence [9]. These factors function as motivators or obstacles to mothers’ practices, with some being under their control and others not. Most studies have primarily concentrated on the issues or challenges that caregivers encounter [10], but there is limited recognition of the solutions that mothers employ to overcome these challenges [11]. The situation is even more dire in agro-pastoral communities, where a problem-lens has mostly been utilised, and the solutions are not well documented [12–15]. A few studies have reported the barriers that agro-pastoral women in African drylands confront and some of the recommended interventions [16–18].

Caregivers’ practices to promote their children’s health and nutritional status are shaped by their underlying knowledge, and the environmental and social factors discussed above influence their room for manoeuvre. Focusing on actual practices not only highlights the challenges but also context-specific actions. This reveals the active role of caregivers in child nutrition and their agency in navigating challenges.

A focus on actors’ knowledge and practices, such as caregivers’ opportunities and possible solutions to challenges being faced, calls for participatory approaches such as photo voice and participatory mapping [19, 20]. These methods and findings inform the design of community-led interventions. In Sierra Leone, a qualitative formative research process, including participatory activities such as the use of diagrams, vignettes, picture discussion cards, songs, games, and storytelling, revealed the significant role of grandmothers in maternal decision-making and practices. This insight led to the inclusion of grandmothers in intergenerational forums as part of the intervention design, which contributed to positive breastfeeding and complementary feeding practices compared to control sites [21, 22]. Another study conducted in Nepal used qualitative methods during its formative research to explore caregivers’ practices, factors that enabled those practices, barriers they faced and existing knowledge gaps. The findings were then used to develop a context-specific behaviour change communication strategy to improve infant and young child feeding practices [23].

The positive-deviance approach has also been used where practices identified in successful caregiving households have been replicated in others to improve health and nutrition outcomes [24–26]. A study conducted in Vietnam used the positive deviance approach where they identified successful caregivers whose children were well-nourished despite poverty. The positive deviant practices were then adopted by other caregivers whose children were malnourished, leading to an 85% decrease in malnutrition [27].

Co-design and co-creation of nutrition interventions, particularly in rural and low-resource settings, remain limited but show great potential for improving health and nutrition outcomes [28, 29]. One example comes from a study addressing iron deficiency anaemia among adolescent girls in four low and middle-income countries: Tanzania, Madagascar, the Philippines and Sri Lanka. Using a co-design process, girls and community members collaboratively designed the intervention, in which Girl Scouts disseminated specific proper nutrition messages such as the promotion of intake of iron-rich foods and their relation to menstruation [30].

Despite the wide recognition that these people-centred approaches better inform sustainable nutrition interventions [31], their application remains limited, particularly in dryland areas. In northern Kenya, the NAWIRI project incorporated qualitative methods in its formative research [18, 32, 33] and a study by Pelto et al. used a focused ethnographic approach to learn about child nutrition practices [34].

Recognising the practices and innovations of actors such as caregivers and incorporating them into context-specific interventions can make those interventions more relevant, applicable, and effective for the target population. The aim of this study is therefore to gain an understanding of (agro)pastoral caregivers’ knowledge of child nutrition and care, including their routine and problem-solving actions, that contribute to the wellbeing and nutritional status of their children.

## Methods

### Activity knowledge analysis

Mothers are primarily responsible for the care of their children and serve as a focal point for promoting their health and nutritional wellbeing. This caregiving system can be understood as a human activity system; one that is shaped by caregivers’ goals, and the actions they undertake to achieve them. These actions are guided by their knowledge, skills and lived experiences. However, they face various challenges, some within their control and others on which they have limited or no influence.

Information about mothers’ activities and the underlying knowledge can be obtained through methods using second-order cybernetics. The concept of second order cybernetics [35] emphasises the interaction between the observer and the observed. It recognises that there is not a single reality, but that what is observed depends on the observer, hence the reality results from the relationship between the subject (observer) and the observed. By examining observers’ perceptions, we can gain insight into how these influence their actions and knowledge and ultimately shape their reality. Therefore, in order for researchers to understand what is important to caregivers’ actions, they need to see the ‘system’ through the caregivers’ eyes.

In a previous study, Kaufmann [36] developed a method using second-order observation to understand the rationale behind the actions of system managers. Building on this, Restrepo et al. [37] developed a participatory tool to conduct the Activity Knowledge Analysis (AKA) that can be used in group discussions to systematically identify routine and problem-solving activities (further information on the process is found below). They used it to understand how dairy farmers achieve high milk quality and how they cope with challenges. This concept and tool have been applied in this study to understand caregivers’ knowledge and innovations in childcare and nutrition.

## The study setting

The study was conducted from January to June 2023 in Saku and Laisamis sub-counties of Marsabit County in northern Kenya. Marsabit County is the least populated county with approximately 459,785 people as of the 2019 census in Kenya, yet the second largest by land area, 70,944 km^2^.

Saku is located in the highlands at 1707 m above sea level, and Laisamis is in the lowlands at 300 m above sea level. The county is inhabited by different ethnic groups who practice varied livelihoods. In this study, we purposively selected Borana and Burji from Saku and Rendille from the lowlands of Laisamis. Borana and Burji communities in Saku are agro-pastoralists and crop farmers, respectively. Other sources of livelihood are trade, casual labour, and self-employment. The Rendille community majorly practice pastoral livestock husbandry.

Saku and Laisamis sub-counties were purposively selected to represent geographic and socio-cultural diversity within the county. These two areas represent distinct agro-ecological zones and are predominantly inhabited by different ethnic communities, each with unique livelihoods and cultural practices. Given resource constraints, it was not feasible to include all four sub-counties in the study. However, the selection is comparable to other regions in the County. Marsabit Central, where Marsabit town is located, shares key characteristics with Moyale and its close surroundings, while Laisamis is comparable to the lowlands of North-Horr inhabited by the Gabra ethnic community.

In Marsabit County, health and nutrition services are delivered through a decentralised system under the leadership of the county government. However, limited infrastructure and resources, particularly in the remote lowland areas, continue to constrain access to essential services. Primary healthcare is largely facilitated by community health promoters and community health assistants, who play a key role in service delivery in the communities. At the county level, Nutrition Technical Forums provide a platform for coordination between government departments and development partners, helping to align ongoing programs and foster collaboration across sectors, including agriculture.

## Study design and sampling

This research, executed under the collaborative project ‘Enhancing women’s agency in navigating changing food environments to improve child nutrition in African drylands’ (NaviNut), utilised a transdisciplinary approach to integrate local and scientific knowledge. Launched in 2021, the project targeted caregivers and children under five years of age. Interactive sessions were conducted with individual women and women’s groups in pastoral and agro-pastoral communities in Marsabit County between January and April 2021. This exploratory phase aimed to understand women’s childcare and nutrition requirements through informal gatherings, narrative interviews, and participatory observations to establish trust and rapport with the participants. A total of nine women’s groups were purposively selected from both rural and peri-urban areas within the two study sites, Saku and Laisamis. These groups represented the Borana, Burji, or Rendille communities. Each group included women caregivers of mixed ages and varying levels of experience in child nutrition. This diversity was intentionally sought to facilitate collaborative learning, a key component of transdisciplinary research. Most women in Marsabit County belong to a self-organised group for various purposes, such as self-help groups or to promote and maintain their cultural practices. Therefore, the women in these groups were a good representation of the wider population.

This current study was conducted from January to July 2023. Here, we first targeted three of the above rural women’s groups representing the Borana, Burji, and Rendille ethnic communities. In addition, we wanted to include the perspectives of mothers who were known to be particularly knowledgeable in the community. The Borana and Rendille groups each recommended three knowledgeable grandmothers. In contrast, the Burji group indicated that the knowledgeable caregivers were already part of their existing group. Therefore, to ensure broader representation, a second Burji group from the peri-urban areas of Saku and a peri-urban Rendille group were included.

Hence, in total, five existing women’s groups participated in the data collection sessions. Each group consisted of 10–12 members attending the sessions. In addition, we included two separate groups comprising three knowledgeable Borana and three knowledgeable Rendille caregivers. We held sessions with the Borana knowledgeable mothers at the beginning of the study and with the Rendille knowledgeable mothers at the end. In the latter case, the researchers presented key findings from the Rendille group sessions to the knowledgeable mothers for their feedback, additional information and insights, and to validate the information collected earlier.

Throughout the research, we worked closely with research assistants who were fluent in the local languages and also built close relationships with the women. They supported with translation during fieldwork activities.

## Data collection

**Fig. 1:**
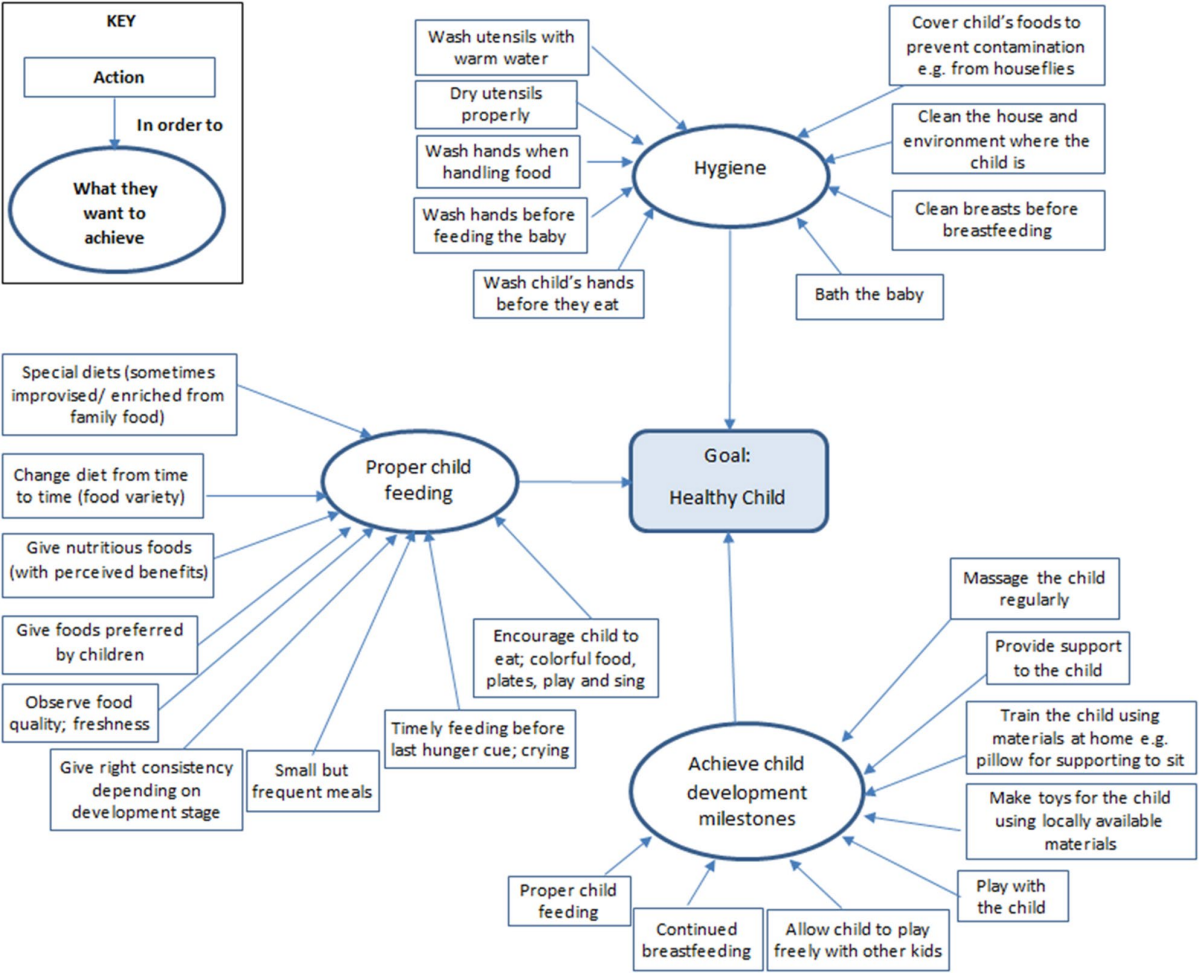
Routine actions on child nutrition and care (6–23 months of age). Source: FGD with Borana, Burji and Rendille

**Fig. 2:**
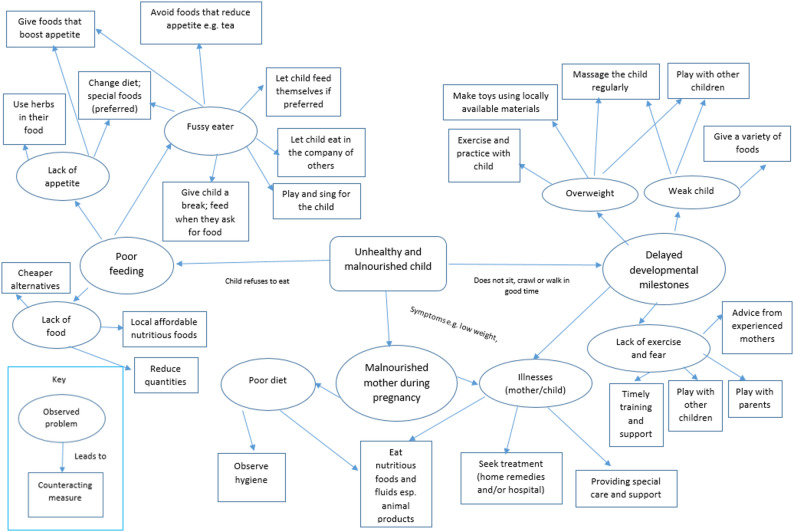
Challenges and respective problem solving actions on challenges related to child nutrition and care. Source: FGDs with Borana, Burji and Rendille.

We used an Activity Knowledge Analysis, a data collection and analysis tool. The tool was conducted in group discussion sessions as follows: caregivers first agreed on the overall goal of having a healthy and well-nourished child, and then shared their aims, which contributed to achieving the overall goal. These aims were noted down. Thereafter, for each aim, the group outlined the actions they undertake to achieve it. For some actions, caregivers explained their observations to assess their progress towards their aims. To identify problem-solving actions, participants first identified the challenges they encountered in child care and nutrition practices. For each challenge, they discussed the perceived underlying causes and the specific actions taken to address or mitigate its effects. Facilitators documented participants’ input in real time on flip charts, similar to a mind map diagram (see Figs. 1 and 2), to show connections between, for example, aims and actions. At the end of the session, facilitators reviewed the points captured to allow participants to add any missed or overlooked contributions. This process allowed for group reflection and confirmation of the shared insights. Eighteen sessions were conducted (16 sessions with the 5 existing women’s groups and 2 sessions with the 2 groups of knowledgeable mothers), each lasting between 1.5 and 3 h. The length and number of sessions depended on the points raised by the groups and their availability.

### Data analysis

The FGD sessions were audio-recorded and transcribed by two research assistants; one fluent in Borana and the other in Rendille. The information documented on flip charts during the sessions, including lists and diagrams outlining aims and routine practices, challenges and problem-solving actions, guided the qualitative analysis by providing initial codes, such as actions, aims and challenges. Additional codes and sub-codes were inductively identified from the transcripts. We used MAXQDA 2022 (VERBI software, 2021) to systematically code the data and retrieve quotations.

## Results

Information obtained from the three ethnic groups was largely similar. Only relevant, subtle differences are highlighted to show specific actions that were more prominent in a particular community.

## Routine actions

The main goal of all mothers is to have a healthy child. To achieve this, mothers are doing some day-to-day activities, classified as routine actions. These actions are informed by knowledge passed down from mothers and grandmothers, practices observed while growing up, and insights gained from peers, other community members, and health workers encountered in hospitals or during home visits.

Some of the routine actions undertaken to promote the health and nutritional status of a child can be grouped into (i) observing hygiene, (ii) achieving developmental milestones, and (iii) proper child feeding.

The specific routine actions within each category are compiled in Fig. 1. This diagram is read from the outside moving inside. The mothers are performing specific actions to achieve some of their aims, which lead to the achievement of the main goal.

Mothers maintain hygiene through a variety of actions on the baby, the mother, the food and the child’s environment. Not all actions are implemented by all mothers but a combination of some of them depending on their needs and conditions. Optimal hygiene practices are only possible when there is sufficient water but during drought, water shortages make this challenging, and some actions are foregone. Children are bathed regularly and in some cases, special herbs (such as fenugreek among Borana and Burji) are added to the water when it is boiled, which can be used for bathing or wiping the child. This helps with the removal of undesirable odours and is believed to have medicinal properties.

To attain a child’s developmental milestones, including sitting, crawling and walking, mothers typically provide their children with nourishing food, massage them and actively participate in providing support or training.

*“For me even before they start moving on their own*,* I turn the table upside down [and] place some clothes*,* and then I place the baby inside and they sit comfortably. When they are strong enough and want to move around*,* I place chairs and stools around for the child to support himself and then I allow the child to play freely and move around the house. The baby pushes these plastic chairs and follows them around. That is how he grows strong and begins to walk on their own” (Burji mother*,* AKA-RA > 6 months).*

Mothers play with their children, allow them to play with other children, and provide toys that encourage play and engagement.

*“…the mother plays with the baby and again buy them toys to play with. The ones who are not [financially] able can use locally available materials to make toys for example cutting a plastic jerry can into half*,* a rope is tied and the baby sits in and gets pulled on the ground like a vehicle.” (Burji mother*,* AKA-RA > 6 months).*

Mothers engaged in the practice of massaging their infants as a means of observing their bodies. It was emphasised that massaging was of great importance for a number of benefits. One of the benefits of massaging was to strengthen the bones, which in turn facilitated the child’s ability to sit, crawl, stand, and walk. Furthermore, massaging is perceived to help shape certain body parts, detect injuries that may otherwise go undetected, promote relaxation, and a good sleep.

*“Massaging also helps to get rid of fatigue*,* if the fatigue is too much the baby will cry*,* and when there is no fatigue the child loves being massaged and they can even laugh. When they are taken good care of as we explained the child will develop a smart brain and grow strong bones.” (Burji mother*,* AKA-RA > 6 months).*

To ensure proper feeding, mothers continue breastfeeding beyond six months and feed their children in a timely manner responding to their hunger cues such as reaching out for food or the breasts avoiding late hunger cues such as crying. Additionally, they observe certain characteristics of the food including texture, taste, and freshness, to assess its quality and appropriateness. The quality and nutritional value of the food are also evaluated based on its effect on the child.

*‘This porridge helps the child to be stronger.’ (Burji Mother*,* AKA-RA > 6 months).*

Consequently, children are fed on special diets, such as those enriched with milk or animal fat, which are believed to enhance their health and nutritional status.

*“A child’s meal is prepared differently from adults. For example*,* tea for kids must contain a lot of milk compared to adults*,* they just use the tea to consume the pancake. When I go to the market*,* I always buy some fruits especially bananas because it is cheaper compared to others.” (Borana Mother*,* AKA-RA > 6 months).*

## Problem-solving actions

Mothers stated that they often encounter problems in their efforts to enhance the health and nutritional status of their children. These problems encompass a range of concerns, including inappropriate feeding practices, malnutrition, delayed developmental milestones, poor hygiene and childhood illness (Fig. 2**)**. They know a number of problem-solving actions for these challenges, which are presented in the figure alongside the observed problems and their underlying causes. For example, fussy eaters tend to eat poorly and therefore mothers have to devise ways to encourage the child to eat.

*‘In such a case [poor feeder]*,* we play with the baby as you feed them. Making them happy as they feed improves how they eat. Some [caregivers] force the baby to eat and that is very wrong. The mother has to play with the baby as she feeds him’ (Borana Mother*,* AKA-PSA > 6 months).*

Observing hygiene was reported to be an important action for preventing illnesses and enhancing the health of a child. However, there are challenges with water scarcity during drought, which mothers have to manoeuvre and still observe some key practices.

*‘We use it sparingly*,* for example*,* the water you use to bath the baby*,* can be used to clean the clothes. We also have jerry cans one for cleaning and the other for cooking. The other way is by skipping some days after washing clothes to minimize wastage’ (Burji Mother*,* AKA-PSA > 6 months).*

**Fig. 3:**
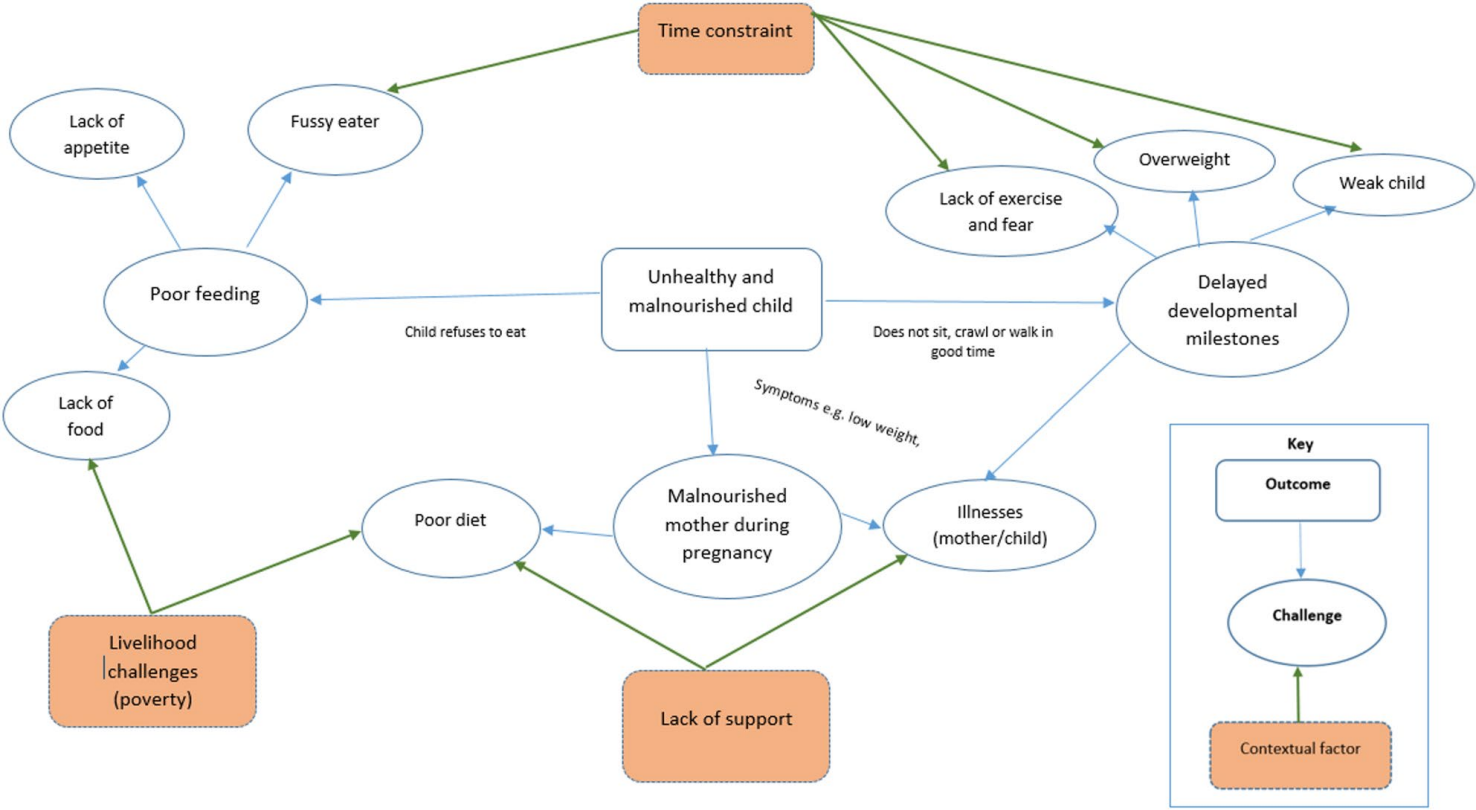
Challenges experienced by mothers when caring for children and contextual factors contributing to their magnitudes. Source FGDs with Borana, Burji and Rendille

The extent to which mothers observe these problems and can implement counteracting or problem-solving actions is contingent upon their household conditions. Three factors were identified by the mothers: livelihood challenges (poverty), lack of support, and time constraints **(**Fig. 3**)**.The influence of these factors is explained below:

### Livelihood challenges

Lack of money or poverty was always mentioned as a pressing challenge for most mothers hindering them to implement the different actions such as providing nutritious child foods, seeking treatment from the hospital in case of illnesses, getting extra help to relieve mothers’ time and accessing water and soap in order to observe proper hygiene practices.

As alleviating or problem solving actions, mothers buy some food items on credit, borrow some money, and diversify their sources of income so as to afford these foods. Other solutions include; borrowing foods from their neighbours, community taking food items to share with the less fortunate especially if they have an infant, mothers purchasing more affordable but less preferred foods or substitute some of the foods with cheaper alternatives. They also prioritize children and buy what they prefer and reduce the quantity of other items. Other items or equipment that could have been useful for child foods such as a blender to improve consistency are expensive and so mothers modify this and use a sieve and press using a spoon.

Family planning and proper child spacing was seen to be important to ease the burden on mothers and promote child’s growth. Drought also affects the produce from the farm and livestock which are major sources of food and income for these households thus making nutritious foods more inaccessible for them. Both are mirrored in the following quote from a Borana mother in the rural parts of Saku.

*“In the past*,* a mother who has given birth is taken care of just like the baby and stays in seclusion for forty days. Animals are slaughtered for her and the most nutritious food is prepared for the forty days she is in seclusion. We did not have to look for fruits for the child because the mother is well fed and the child can get enough breast milk that is healthy for them. Initially*,* we had great produce from our farms but now due to prolonged drought we barely get produce from our farms*,* what we are discussing now is what the child is supposed to have (good for a child) and not what we are giving them. Even if we cannot provide we know what is good for a child’s health*,* so if we were able we would give them these good meals.” (Borana Mother*,* AKA-PSA > 6 months).*

Mothers are engaged in socio-economic activities such as caring for livestock and farming activities. However, in recent decades, they have increasingly faced challenges during droughts, with water and pasture being scarce and far from their homes. This has resulted in the movement of the main herd to distant places. In particular, during such periods, the market value of livestock tends to be low, while prices for crops and vegetables tend to increase. Depending on the number of other tasks and the herd size, there may be a need for additional labour, which may not be available for free. Furthermore, costs are incurred for vaccinations and the treatment of animals. In order to manage their resources, households are forced to reduce their livestock numbers and use the proceeds to purchase other food and meet household and family expenses. Alternatively, they engage the services of another individual to care for the animals and/or consult with their husbands to determine the most effective division of labour.

### Time constraint

Mothers face time constraints due to multiple competing tasks, which limits the time available for child care. Some of these tasks include: household chores such as cleaning, cooking, fetching firewood and water, as well as socio-economic activities like caring for livestock, agricultural activities, trade and casual labour. A major problem with these activities in the drylands is that they require people to walk long distances to get supplementary feeds for livestock, water for domestic use and firewood for cooking. Mothers are also increasingly being relied on economically, hence they engage in economic activities to generate some income, which further strains their already limited time. Domestic conflict worsens the situation, as it can result in mothers not receiving financial or instrumental support (practical help with tasks) from their husbands.

Strategies to overcome these challenges include keeping livestock closer to the home, which is not possible during droughts. Mothers who cannot afford extra help and are involved in economic activities, take their children to work so that they can still care for and feed them. If this is not possible, mothers prepare meals for their children in advance, which are then fed to the child by the person who is left looking after them. As for broken marriages and family conflicts, mothers need to seek support from their immediate family, such as their mother or sister.

### Lack of support

Mothers are supported by their spouses, family members and the community in many ways that reduce the burden of childcare. While a larger proportion of mothers receive only financial support, others receive both financial support and help with household tasks. A smaller proportion receive no support and have to divide their time between household tasks and income-generating activities. Help with housework and childcare, or ‘instrumental support’, is usually lacking due to the prevailing attitude that this is not a man’s job. Other husbands are often out of the house due to work commitments. Some men are unable to support their families financially due to unemployment or extramarital affairs, which puts a strain on their finances. Family conflicts were also reported to aggravate the situation. Women prefer to receive some financial support and will understand if their husbands are too busy to help with some work at home. They are also encouraged to be patient if the man has no source of income at the moment. Without financial support, mothers look for casual work to earn some income. They sell household livestock without the husband’s consent and seek support from elders to convey the message to the husband and create an understanding of their needs. In some cases, they are also supported by relatives. Husbands who are reluctant to help with some tasks need to be counselled and their wives can talk to them.

The Rendille knowledgeable mothers agreed with all the practices reported by the mothers and added some specific home remedies, such as using herbs to stimulate children’s appetite. They also mentioned that men could support their wives in livestock-related activities by hiring someone to help them and compensating them with a goat. This would give mothers more time to care for their children.

### Sub-optimal practices and Myths

Despite the overall reporting of useful practices used routinely or to mitigate specific challenges, the following suboptimal practices and myths emerged:

- Force feeding: Some mothers may use discouraged practices such as force feeding the child. This could result from time constraints limiting the possibility for responsive feeding.
- Sheep meat: Among Borana mothers, there is a belief that consumption of sheep meat before the age of two may cause stuttering or delayed speech.
- Eggs: Within a specific clan of the Rendille community, the consumption of eggs by children is prohibited due to cultural restrictions.
- ‘Bad eyes’ and appetite loss: Borana mothers expressed the belief that if a child is looked at with ‘bad eyes’ (envy or ill intent), it can negatively affect the child’s appetite and overall health. The remedy was to mix locally prescribed herbs into the child’s food.
- ‘Bad teeth’: In some cases, infant milk teeth were believed to emerge at an inappropriate time, hence delaying development and worsening the child’s health and nutritional status. The only solution in this case was to have these teeth removed by a local specialist.

Intercultural exchange may help demystify these practices by showing caregivers that approaches used by other groups can also support child well-being, since they were specific to an ethnic community.

## Discussion

This qualitative study used the activity and knowledge analysis method [36, 37], offering a participatory approach to examine (agro)-pastoral caregivers’ knowledge and practices related to child care and nutrition. This method allowed for an in-depth exploration of their routine actions, the challenges they face, and their problem-solving actions in their specific contexts, to improve the health and nutritional well-being of their infants and young children.

Conventional methodologies like Knowledge, Attitudes, and Practices (KAP) surveys have shown associations between caregivers’ nutritional knowledge and child practices and nutritional status in Kenya [38, 39]. These surveys are typically conducted with large sample sizes in a relatively short period, offering insights into the distribution of knowledge across populations. However, their reliance on standardized, structured questions limits the depth of exploration of caregivers’ knowledge and practices, potentially overlooking the contextual challenges, decision-making processes and local problem-solving strategies.

For instance a KAP survey conducted across all four sub-counties in Marsabit County (Saku, Laisamis, Moyale and North Horr), found that most caregivers had low knowledge about the age-appropriate introduction of solid foods and poor complementary feeding practices [40]. This was concluded based on responses to closed questions such as the recommended age to start complementary feeding, reasons for giving complementary foods at 6 months, the right consistency for a child’s food, ways to make foods more nutritious, and ways to encourage young children to eat.

In contrast, our study used a participatory approach and engaged a smaller number of caregivers with whom we had built trustful relationships over time. It allowed for in-depth discussions of their actions and their underlying knowledge in caring for their children. This approach revealed the many different options available to mothers from the Rendille, Burji, and Borana communities; knowledge that cannot be fully captured by the structured scoring systems of the KAP survey. Indeed, mothers reported various child-feeding practices, such as providing children with nutritious meals of the right consistency, frequently and on time, in line with WHO recommendations [41]. They also report offering foods that children prefer and that they believe are beneficial for their growth and development, an outcome that depends on good nutrition from an early stage [42, 43]. Mothers reported using a variety of techniques to encourage their children to eat, demonstrating a loving relationship during feeding times. Such responsive feeding practices and affectionate interactions promote healthy dietary behaviours and facilitate child development [44].

Certain practices respond clearly to the contextual conditions, for example, the Rendille women often enriched their children’s diets with locally available animal fat, a common practice in this pastoral community. All communities also used milk to enrich child foods and other ingredients depending on their availability and affordability. This practice has also been reported among the Gabra and Pokot agro-pastoral communities in Kenya [34, 45]. Due to water scarcity, mothers used local herbs for bathing to remove unpleasant odours.

In other parts of the world, mothers use special toys, dolls and pillows to help their children sit and walk. For example, to encourage a child to walk, they might provide toys that can be pushed. However, the mothers in this study did not have access to such items and therefore used household materials such as washbasins, cloths as pillows, upside-down chairs, push or pull jerry cans and tables to support their children’s sitting and walking development, reflecting the creativity in their caregiving practices. Physical and emotional support has been associated with positive outcomes in children’s brain development and cognitive functioning [46].

The problem-solving activities highlight the challenges mothers face and the actions they take to address them (see Fig. 3). Mothers in pastoral areas of the Somali region in Ethiopia, Uganda, and Rwanda have also highlighted barriers, such as low accessibility & affordability of diets, time constraints and limited autonomy, to optimal nutrition, especially around child feeding [14, 47, 48]. Challenges such as weak or sick children and poor or fussy eaters require particular knowledge, and in this case, experienced older mothers would provide informational support to the young mothers. Grandmothers have also been recognized in studies conducted in Sierra Leone, Malawi, and other parts of Kenya for their informational and practical support to mothers in matters related to child care and nutrition [21, 49–51]. Overcoming these challenges often means spending more time with the child. This is difficult for mothers who are involved in multiple activities with little or no support from family members.

Overall, poverty, time constraints and lack of support from other family members emerged as important contextual factors influencing whether mothers face these challenges. Challenges such as poor diets, due to lack of food or nutritious ingredients, are mainly caused by poverty. Particularly in the pastoral areas during droughts, almost all families are confronted with this temporal poverty. Poorer households are pushed further into poverty especially during recurrent droughts [52]. Poverty is a barrier to ensuring children’s health and nutrition as mothers usually need income to buy nutritious food, seek health care or maintain hygiene [53]. Our study found that, similar to mothers in Rwanda, Ghana and Senegal [7, 8, 54], mothers resort to various strategies including borrowing money and eating less preferred but cheaper foods to meet their children’s needs.

Mothers often face the challenge of managing multiple responsibilities with limited time [10, 55, 56]. This is evident in the drylands, where labour demand increases, especially during droughts, as women have to walk longer distances to fetch water and firewood and for livestock-related activities [57, 58]. About half of mothers reported engaging in economic activities to support their families. When income is provided solely by their husbands, some mothers reported having limited decision-making power over financial and health matters. Mothers in this study acknowledged the importance of child spacing and family planning as strategies to ease both physical and financial burdens. Marsabit County ranks among the top six counties in Kenya with the highest unmet need for family planning (37.6%). Contributing factors include negative perceptions of modern family planning methods and limited male involvement and support [3, 59]. Similar perspectives have been reported among mothers in Uganda, who recognized the value of family planning despite limited support from their spouses [47]. An intervention study in Tanzania further reinforces this point, highlighting the benefits of integrating family planning with nutrition services at health clinics [60].

Efforts to adopt optimal caregiving practices were also hindered by family conflicts, a barrier similarly observed in rural Ethiopia, where household disputes and domestic violence negatively impacted infant and young child feeding [11]. Overall, limited autonomy continues to constrain caregivers’ confidence and their ability to effectively manage challenges or implement optimal practices [61, 62].

In contexts of poverty and time constraints, social support from partners, family members, and community members becomes crucial. Social support networks play a pivotal role in supporting mothers’ desired practices and building their confidence. Our study has shown the importance of social support in helping mothers implement desired practices such as preparing nutritious meals, feeding their children on time, providing emotional support, and monitoring their children to prevent accidents and injuries. The effectiveness of social support networks in improving caregivers’ knowledge, confidence, perceptions and child-feeding practices has also been documented in Western Kenya [63, 64]. Leveraging existing social support networks within communities can further strengthen support for mothers and improve their agency, ultimately contributing to improved maternal and child health outcomes.

In most countries in sub-Saharan Africa, Non-Governmental Organizations (NGOs) or government agencies implement interventions to improve infant and young child nutrition. Nutrition education is a common intervention, either as a stand-alone or as part of a broader package. In Kenya, nutrition education interventions, including health talks and counselling, are delivered in health facilities and at the community level [65], using the social behaviour change communication strategy [66]. These interventions are in line with global maternal and child nutrition guidelines and aim to promote optimal breastfeeding, appropriate complementary feeding practices and growth monitoring.

During health talks and counselling sessions, health workers share key messages with caregivers to improve their child nutrition practices. Our study shows that mothers are knowledgeable about most of these topics. Therefore, when they are taught using these standard key messages, they are likely to feel that their knowledge is being disregarded and that their specific conditions are being ignored. A study conducted in another region in Kenya highlighted caregivers’ appreciation for practical counselling sessions and for counsellors who demonstrated respect and accommodated them accordingly. Conversely, poor communication from counsellors, e.g. with regard to the need for feeding certain foods, was reported to contribute to negative caregiving practices, such as forceful feeding [67].

It is important to build on what caregivers already know and spend more time sharing practical information on aspects where they have limited or no knowledge. While these key messages are important, they must be tailored to the specific contexts of communities. For example, one of the key messages for lactating mothers and young children is to consume a variety of foods from the different 7–10 food groups. However, in Marsabit County, dietary diversity is largely dependent on food availability and accessibility, which can vary between locations even within a relatively small part of the drylands of Kenya [68] for a variety of reasons, including ecological conditions and market infrastructure. It is therefore important to provide practical examples of foods that are accessible in the different seasons. Encouraging young mothers to use traditional foods, including wild vegetables and fruits, would be helpful in complementing diets based on home-produced and market foods, with the latter being the main source during dry seasons and droughts.

In addition, practical lessons such as cooking demonstrations can help them gain knowledge from older women on how to prepare nutritious traditional foods based on readily available ingredients. An intervention study conducted in Isiolo and Marsabit counties provided counselling to caregivers on food preservation and the consumption of traditional foods, drawing on practices already present within the community. This approach led to increased dietary diversity [69]. Integrating such community-based efforts into Kenya’s existing community health system structure could enhance the delivery and sustainability of nutrition programs, particularly when these programs are context-specific and driven by local knowledge and priorities.

Mothers in northern Kenya typically evaluate the quality of food based on its effects on their children’s health and nutritional status rather than its nutrient composition, as they usually do not have information on the specific nutrients in each food item [68]. It is therefore more useful to share information based on the observable effects of foods rather than their nutrient content. This can be achieved by contextualising and updating existing nutrition education materials to reflect local knowledge and practices. Additionally, enhancing peer-to-peer learning through established women’s groups offers an avenue for culturally relevant knowledge exchange. A nutrition-sensitive agriculture program in India effectively used storytelling, a participatory method, to deliver behaviour change messages to women’s self-help groups. The women were more engaged when the stories resonated with their own experiences, addressed emotionally relevant topics such as gender-related issues, and when facilitators shared personal examples from their own lives. This highlights the importance of participatory and culturally relevant communication strategies and the effectiveness of these messages, especially when delivered through trusted community structures [70].

Key messages related to hygiene also need to be assessed for their applicability in the dryland context. For example, it is recommended that mothers wash their hands with soap and running water at critical times (e.g. before preparing food, after changing a child’s nappy, after caring for a sick person, etc.). In a dryland environment such as the lowlands of Marsabit County, where water insecurity is prevalent [58], and water must be carried in 20-litre jerry cans from wells that are often 3–10 km or more away, adhering to this recommendation poses a significant challenge. In addition, poorer households cannot afford to buy soap regularly. In such cases, community members can suggest cheaper and practical alternatives, such as recycling leftover soap pieces or using ashes, which is already a common practice.

People-centred nutrition interventions, which actively involve the ‘end-users’ throughout the process from problem identification to solution design, gain importance [31]. Although intensive and time-consuming, they tend to be more effective and sustainable. To achieve this, specific methodologies need to be adopted, such as participatory techniques. For instance, an intervention targeting Nepali pregnant women employed a participatory learning and action (PLA) cycle, where women first identified challenges, then developed and implemented community-supported strategies, and finally reviewed outcomes and next steps. The integration of food and cash support further amplified the effectiveness of this approach, resulting in increased consumption of animal-source foods and iron-folate supplements, alongside measurable improvements in nutritional status [71]. It is therefore important to first understand the different actions that caregivers routinely take or use to overcome challenges, and to identify knowledge gaps. Gaining such insight into caregivers’ abilities and strategies can help inform and design context-specific interventions that are more relevant, feasible, and likely to be adopted by mothers.

### Strengths and limitations of the approach and directions for further research

The Activity Knowledge Analysis (AKA) method is geared towards getting a shared understanding among participants and researchers on routine and problem-solving practices used in an activity system, such as the caregivers’ child-rearing system. One of its strengths lies in its ability to identify practices that are effective within specific contextual conditions (routine actions). Secondly, it allows for the identification of challenges for which caregivers currently have effective solutions (problem-solving actions). These actions have proven useful and are known and used within the community. They can be considered local best practices and can be utilised for further counselling and peer learning. However, caregivers may lack effective solutions for some challenges, which highlights areas for further research or targeted interventions.

However, the AKA method does not allow for the identification of who uses specific practices or how widely those practices are adopted within the community. Due to its participatory nature, the number of respondents is relatively small, making it important to ensure adequate representation of caregivers with diverse circumstances and experiences. Furthermore, because the method focuses on practices that align with the intended goals of the activity system, it usually does not capture suboptimal practices; those used either due to a lack of knowledge or as a necessity during certain periods (e.g., during drought), despite awareness of better alternatives. Nevertheless, such practices can become apparent when caregivers discuss their respective practices and compare them with what they do under specific circumstances.

To better understand the distribution and variability of such knowledge, future studies could incorporate in-depth individual interviews. It would also be valuable to include a broader range of actors playing a role in caregiving such as fathers and community health workers.

## Conclusion

Mothers play a pivotal role in their children’s health and well-being, taking various actions to ensure optimal nutrition, hygiene and development. From establishing eating habits to promoting cognitive and physical growth through play, their role is vital.

Despite their efforts, mothers face challenges such as feeding difficulties, malnutrition and delays in developmental milestones, often worsened by contextual factors such as poverty, limited support and time constraints. In response, they adopt measures such as dietary adaptations and income diversification, highlighting their resilience during difficulties. Social support emerges as a crucial buffer, easing the mothers’ tasks and enabling them to prioritise childcare.

This study demonstrates the complex interplay of various factors, including cultural, social, economic and environmental, that influence maternal behaviour and child health outcomes. This highlights the need for a multifaceted approach that takes into account socio-economic factors and strengthens support systems to empower mothers to effectively promote the wellbeing of their children. This could include integration of livelihood support programs with nutrition interventions, for example, by strengthening self-help groups and improving access to financial resources to enhance food systems through sustainable production, processing and value addition. Recognising their skills and co-creating these solutions is critical to further empowering their agency. By recognising and addressing these complexities, we can establish a foundation for improving current programs with context-specific interventions that promote maternal and child wellbeing.

## Data Availability

No datasets were generated or analysed during the current study.

## Abbreviations

ASALs: Arid and Semi-Arid Lands, KAP: Knowledge, Attitudes and Practices
FGD: Focus Group Discussions
AKA: Activity Knowledge Analysis
WHO: World Health Organization
NGOs: Non-Governmental Organizations

## References

[1] UNICEF. Levels and trends in child mortality [Internet]. 2021 Dec. Available from: https://data.unicef.org/resources/levels-and-trends-in-child-mortality/

[2] WHO. Malnutrition- Key Facts [Internet]. World Health Organ. 2024 [cited 2024 Dec 1]. Available from: https://www.who.int/news-room/fact-sheets/detail/malnutrition

[3] Kenya National Bureau of Statistics (KNBS), Ministry of Health (MoH), National AIDS Control Council, National Council For Population and Development, The DHS Program, ICF International., Kenya Demographic and Health Survey 2022. Key Indicators Report [Internet]. Nairobi, Kenya and Rockville, Maryland, USA; 2023 Jan. Available from: https://dhsprogram.com/pubs/pdf/PR143/PR143.pdf

[4] Munene F, Integrated. SMART Survey Marsabit County, Kenya [Internet]. Marsabit, Kenya: Concern Worldwide; 2023 Jul. Available from: https://nutritionhealth.or.ke/wp-content/uploads/2024/05/Marsabit-County-July-2023-SMART-SURVEY-REPORT.pdf

[5] Young H. Nutrition in Africa’s drylands: A conceptual framework for addressing acute malnutrition [Internet]. 2020 p. 25. Available from: https://fic.tufts.edu/wp-content/uploads/FIC-malnafricandrylands_8.7.2020.pdf

[6] Bazzano A, Kaji A, Felker-Kantor E, Potts K. Qualitative Studies of Infant and Young Child Feeding in Lower-Income Countries: A Systematic Review and Synthesis of Dietary Patterns and Their Determinants. 2017. 10.3390/nu9101140 PMC5691756 29057842

[7] Monterrosa EC, Pelto GH, Frongillo EA, Rasmussen KM. Constructing maternal knowledge frameworks. How mothers conceptualize complementary feeding. Appetite. 2012;59:377–84. 22698974 10.1016/j.appet.2012.05.032

[8] Ahishakiye J, Bouwman L, Brouwer ID, Matsiko E, Armar-Klemesu M, Koelen M. Challenges and responses to infant and young child feeding in rural rwanda: a qualitative study. J Health Popul Nutr. 2019;38:43. 31831068 10.1186/s41043-019-0207-z PMC6907215

[9] Nabwera HM, Moore SE, Mwangome MK, Molyneux SC, Darboe MK, Camara-Trawally N, et al. The influence of maternal psychosocial circumstances and physical environment on the risk of severe wasting in rural Gambian infants: a mixed methods approach. BMC Public Health. 2018;18:109. 29304780 10.1186/s12889-017-4984-2 PMC5756408

[10] Nankumbi J, Muliira JK. Barriers to infant and Child-feeding practices: A qualitative study of primary caregivers in rural Uganda. J Health Popul Nutr. 2015;33:106–16. 25995727 PMC4438654

[11] Gizaw AT, Sopory P, Sudhakar M. Barriers and coping responses towards infant and young child feeding practices in rural ethiopia: a descriptive qualitative study. BMJ Open. 2023;13:e077008. 37821129 10.1136/bmjopen-2023-077008 PMC10582866

[12] Gebre A, Reddy PS, Mulugeta A, Sedik Y, Kahssay M. Prevalence of malnutrition and associated factors among Under-Five children in pastoral communities of Afar regional State, Northeast ethiopia: A Community-Based Cross-Sectional study. J Nutr Metab. 2019;2019:9187609. 31275645 10.1155/2019/9187609 PMC6589243

[13] Wuneh AG, Ahmed W, Bezabih AM, Reddy PS. Dietary diversity and meal frequency practices among children aged 6–23 months in agro pastoral communities in Afar Region, ethiopia: A Cross-sectional study. Ecol Food Nutr. 2019;58:575–96. 31353951 10.1080/03670244.2019.1644328

[14] Abas AH, Ahmed AT, Farah AE, Wedajo GT. Barriers to optimal maternal and child feeding practices in pastoralist areas of Somali Region, Eastern ethiopia: A qualitative study. Food Nutr Sci. 2020;11:540–61.

[15] Mutuku JN, Ochola S, Osero J. Maternal knowledge and complementary feeding practices and their relationship with nutritional status among children 6–23 months old in pastoral community of Marsabit County, kenya: A Cross-Sectional study. Curr Res Nutr Food Sci J. 2020;8:862–76.

[16] Catley A, Lotira R, Hopkins C. Hidden peaks Women’s knowledge on seasonality and root causes of child malnutrition in Karamoja, Uganda and their programming references [Internet]. 2018 Nov. Available from: https://karamojaresilience.org/wp-content/uploads/2021/05/201811_krsu_hidden_peaks_nutrition_online.pdf

[17] FAO U. Seasonality of malnutrition: Community knowledge on patterns and causes of undernutrition in children and women in Laisamis, Marsabit County, Kenya [Internet]. FAO and UNICEF. 2020 [cited 2021 Feb 23]. Available from: http://www.fao.org/documents/card/en/c/ca8749en

[18] Burns J, Catley A, Mahmoud H. Women’s knowledge on the seasonality and causes of child malnutrition in Marsabit County, Kenya. 2021 [cited 2022 Apr 5]; Available from: https://rgdoi.net/10.13140/RG.2.2.13819.18726

[19] Chappell E, Chan E, Deen C, Brimblecombe J, Cadet-James Y, Hefler M, et al. Using photovoice to generate solutions to improve food security among families living in remote aboriginal and/or Torres Strait Islander communities in Australia. BMC Public Health. 2024;24:785. 38481178 10.1186/s12889-024-18200-x PMC10935805

[20] Sly BC, Weir TL, Cunningham-Sabo L, Leisz SJ, Stull VJ. Increasing household diet diversity and food security in rural Rwanda using Small-Scale Nutrition-Sensitive agriculture: A Community-Engaged Proof-of-Concept study. Nutrients.2023;15:3137. 10.3390/nu15143137 PMC10386443 37513555

[21] MacDonald CA, Aubel J, Aidam BA, Girard AW. Grandmothers as change agents: developing a culturally appropriate program to improve maternal and child nutrition in Sierra Leone. Curr Dev Nutr. 2019;4:nzz141. 31893262 10.1093/cdn/nzz141 PMC6932963

[22] Aidam BA, MacDonald CA, Wee R, Simba J, Aubel J, Reinsma KR, et al. An innovative Grandmother-Inclusive approach for addressing suboptimal infant and young child feeding practices in Sierra Leone. Curr Dev Nutr. 2020;4:nzaa174. 33409444 10.1093/cdn/nzaa174 PMC7773705

[23] Locks LM, Pandey PR, Osei AK, Spiro DS, Adhikari DP, Haselow NJ, et al. Using formative research to design a context-specific behaviour change strategy to improve infant and young child feeding practices and nutrition in Nepal. Matern Child Nutr. 2015;11:882–96. 23557321 10.1111/mcn.12032 PMC6860308

[24] Schooley J, Morales L. Learning from the community to improve maternal-child health and nutrition: the positive Deviance/Hearth approach. J Midwifery Womens Health. 2007;52:376–83. 17603960 10.1016/j.jmwh.2007.03.001

[25] Bisits Bullen PA. The positive deviance/hearth approach to reducing child malnutrition: systematic review. Trop Med Int Health TM IH. 2011;16:1354–66. 21749582 10.1111/j.1365-3156.2011.02839.x

[26] Shonkoff E, Folta SC, Fitopoulos T, Ramirez CN, Bluthenthal R, Pentz MA, et al. A positive deviance-based qualitative study of stress, coping, and feeding practices among low-income, Hispanic mothers whose children do versus do not Meet guidelines for fruit and vegetable intake. Health Educ Res. 2020;35:584–604. 33367771 10.1093/her/cyaa037 PMC8463094

[27] Singhal A, Svenkerud PJ. Flipping the diffusion of innovations paradigm: embracing the positive deviance approach to social change. Asia Pac Media Educ. 2019;29:151–63.

[28] Needham C, Partridge SR, Alston L, Rawstorn JC, Livingstone KM. Co-designing interventions to improve diets in rural communities. Proc Nutr Soc Published online. 2025;1–7. 10.1017/S0029665125000060 10.1017/S0029665125000060 39910988

[29] Singh DR, Sah RK, Simkhada B, Darwin Z. Potentials and challenges of using co-design in health services research in low- and middle-income countries. Glob Health Res Policy. 2023;8:5. 36915174 10.1186/s41256-023-00290-6 PMC10009993

[30] Dyke E, Pénicaud S, Hatchard J, Dawson A-M, Munishi O, Jalal C. Girl-Powered nutrition program: key themes from a formative evaluation of a nutrition program Co-designed and implemented by adolescent girls in Low- and Middle-Income countries. Curr Dev Nutr. 2021;5:nzab083. 34286176 10.1093/cdn/nzab083 PMC8282357

[31] Young S, Klemm R, Baker S. The power of People-Centered nutrition interventions. In: Kraemer K, Cordaro JB, Fanzo J, Gibney M, Kennedy E, Labrique A, Steffen J, Eggersdorfer M, editors. Good Nutrition: Perspectives for the 21st Century. Basel: Karger; 2016. pp. 197–208.

[32] Catholic Relief Services (CRS), Nawiri USAID. Use of Trials of Improved Practices (TIPs) to Improve Complementary Feeding, Including the Consumption of Preserved Foods, in Isiolo and Marsabit Counties, Kenya [Internet]. Nairobi, Kenya: Catholic Relief Services; 2021 Nov. Available from: https://www.advancingnutrition.org/sites/default/files/2023-04/Use%20of%20TIP%20to%20Improve%20Complementary%20Feeding%20Inc%20Consumption%20of%20Preserved%20Foods%20in%20Isiolo%20and%20Marsabit%20Counties%20-%20Final.pdf

[33] Burns J, Catley A, Mahmoud H. Using Participatory Epidemiology to Investigate the Causes and Seasonality of Acute Malnutrition in Marsabit and Isiolo Counties, Northern Kenya: Methods and Experiences [Internet]. Tufts University Nawiri project: Feinstein International Center, Friedman School of Nutrition Science and Policy; 2021 p. 32. Available from: https://fic.tufts.edu/wp-content/uploads/PE_MethodsReport-_9.10.21Final.pdf

[34] Pelto GH, Thuita FM. Focused ethnographic studies of infant and young child feeding Behaviours, Beliefs, Contexts, and environments in Vihiga, Kitui, Isiolo, Marsabit, and Turkana counties in Kenya. Nairobi, Kenya: GAIN; 2015.

[35] Von Foerster H. Observing systems [Internet]. 2nd ed. Intersystems Publications; 1984 [cited 2024 Jun 7]. Available from: https://cir.nii.ac.jp/crid/1130000797933001216

[36] Kaufmann BA. Second-order cybernetics as a tool to understand why pastoralists do what they do. Agric Syst. 2011;104:655–65.

[37] Restrepo MJ, Lelea MA, Kaufmann B. Second-Order cybernetic analysis to Re-construct farmers’ rationale when regulating milk production. Syst Pract Action Res. 2016;29:449–68.

[38] Chege PM, Kuria EN. Relationship between nutrition knowledge of caregivers and dietary practices of children under five in Kajiado County, Kenya. Women’s Health Bull. 2017;4:1–5.

[39] Nkoitoi SN, Chege P, Walekhwa M. Association between caregiver’s nutrition knowledge and nutrition status of children 6 to 23 months: A case study of Narok County referral hospital. Afr J Nutr Diet. 2024;2:10–20.

[40] MOH, County Government of Marsabit., USAID. Marsabit County Maternal, Infant and Young Child Nutrition (MIYCN) Knolwedge Attitude and Practices (KAP) Baseline Survey for Marsabit County [Internet]. 2018 Jan. Available from: https://www.nutritionhealth.or.ke/wp-content/uploads/MIYCN%20Assessments%20Reports/Marsabit%20County%20MIYCN%20KAP%20Report%20-%20January%202018.pdf

[41] World Health Organization (WHO). WHO guideline for complementary feeding of infants and young children 6–23 months [Internet]. Geneva: World Health Organization. 2023 Oct. Available from: https://iris.who.int/bitstream/handle/10665/373358/9789240081864-eng.pdf?sequence=1

[42] Hurley KM, Yousafzai AK, Lopez-Boo F. Early child development and nutrition: A review of the benefits and challenges of implementing integrated Interventions1234. Adv Nutr. 2016;7:357–63. 26980819 10.3945/an.115.010363 PMC4785470

[43] Roberts M, Tolar-Peterson T, Reynolds A, Wall C, Reeder N, Rico Mendez G. The effects of nutritional interventions on the cognitive development of Preschool-Age children: A systematic review. Nutrients. 2022;14:532. 35276891 10.3390/nu14030532 PMC8839299

[44] Pérez-Escamilla R, Segura-Pérez S, Hall Moran V. Dietary guidelines for children under 2 years of age in the context of nurturing care. Matern Child Nutr. 2019;15:e12855. 31240831 10.1111/mcn.12855 PMC7199077

[45] Kimiywe J, Craig H, Agyapong A, Thorne-Lyman A, Matsisa P, Kiige L et al. Diets of infants and young children in two counties of Kenya: key drivers and barriers to improvement. Matern Child Nutr. 202;20;Suppl 3: e13334. 10.1111/mcn.13334 PMC10782136 36468358

[46] Onyango S, Kitsao-Wekulo P, Langat N, Okelo K, Murdock DE, Utzinger J, et al. Maternal stimulation and early child development in sub-saharan africa: evidence from Kenya and Zambia. BMC Public Health. 2023;23:2418. 38053131 10.1186/s12889-023-17235-w PMC10696819

[47] Ickes SB, Heymsfield GA, Wright TW, Baguma C. Generally the young mom suffers much: Socio-cultural influences of maternal capabilities and nutrition care in uganda: Socio-cultural factors that shape nutrition care giving in Uganda. Matern Child Nutr. 2017;13:e12365. 27650794 10.1111/mcn.12365 PMC6866037

[48] Dusingizimana T, Weber JL, Ramilan T, Iversen PO, Brough L. A qualitative analysis of infant and young child feeding practices in rural Rwanda. Public Health Nutr. 2020;1:10. 10.1017/S1368980020001081 PMC10195323 32611464

[49] Bezner Kerr R, Dakishoni L, Shumba L, Msachi R, Chirwa M. We grandmothers know plenty: Breastfeeding, complementary feeding and the multifaceted role of grandmothers in Malawi. Soc Sci Med. 2008;66:1095–105. 18155334 10.1016/j.socscimed.2007.11.019

[50] APHRC. The Role of Grandmothers in Infant. and Young Child Feeding and Care in Kenya [Internet]. APHRC. 2015 [cited 2024 May 25]. Available from: https://aphrc.org/blogarticle/the-role-of-grandmothers-in-infant-and-young-child-feeding-and-care-in-kenya/

[51] Thuita F, Mukuria A, Muhomah T, Locklear K, Grounds S, Martin SL. Fathers and grandmothers experiences participating in nutrition peer dialogue groups in vihiga County, Kenya. Matern Child Nutr. 2021;17(Suppl 1):e13184. 34241953 10.1111/mcn.13184 PMC8269141

[52] Mauerman M, Ross C, Ilboudo Nébié E, Anderson W, Jensen N, Chelanga P. The long-term impact of multi-season droughts on livestock holdings and pastoralist decision-making in Marsabit, Kenya. J Arid Environ. 2023;211:104928.

[53] Chakona G. Social circumstances and cultural beliefs influence maternal nutrition, breastfeeding and child feeding practices in South Africa. Nutr J. 2020;19:47. 32434557 10.1186/s12937-020-00566-4 PMC7240933

[54] Armar-Klemesu M, Osei-Menya S, Zakariah-Akoto S, Tumilowicz A, Lee J, Hotz C. Using ethnography to identify barriers and facilitators to optimal infant and young child feeding in rural ghana: implications for programs. Food Nutr Bull. 2018;39:231–45. 29486585 10.1177/0379572117742298

[55] Athavale P, Hoeft K, Dalal RM, Bondre AP, Mukherjee P, Sokal-Gutierrez K. A qualitative assessment of barriers and facilitators to implementing recommended infant nutrition practices in Mumbai, India. J Health Popul Nutr. 2020;39:7. 32718334 10.1186/s41043-020-00215-w PMC7385866

[56] Burns J, Emerson JA, Amundson K, Doocy S, Caulfield LE, Klemm RDW. A qualitative analysis of barriers and facilitators to optimal breastfeeding and complementary feeding practices in South Kivu, Democratic Republic of congo. Food Nutr Bull. 2016;37:119–31. 27053492 10.1177/0379572116637947

[57] Galwab AM, Koech OK, Wasonga OV, Kironchi G. Gender-differentiated roles and perceptions on climate variability among pastoralist and agro-pastoralist communities in marsabit, Kenya. Nomadic Peoples. 2024;28:41–71.

[58] Balfour N, Obando J, Gohil D. Dimensions of water insecurity in pastoralist households in Kenya. Waterlines. 2020;39:24–43.

[59] Matoke VO, Gitonga EM, Owaka IO, Okari GM, Mutabazi M, Ogutu GM, et al. Influence of male targeted short message service on knowledge, nature of attitude and male involvement on uptake of family planning among spouses in Marsabit County, Kenya. Int J Community Med Public Health. 2024;11:4198–204.

[60] Cooper CM, Kavle JA, Nyoni J, Drake M, Lemwayi R, Mabuga L, et al. Perspectives on maternal, infant, and young child nutrition and family planning: considerations for rollout of integrated services in Mara and Kagera, Tanzania. Matern Child Nutr. 2019;15(Suppl 1):e12735. 30748120 10.1111/mcn.12735 PMC6593746

[61] Salinger AP, Vermes E, Waid JL, Wendt AS, Dupuis SJN, Kalam MA, et al. The role of self-efficacy in women’s autonomy for health and nutrition decision-making in rural Bangladesh. BMC Public Health. 2024;24:338. 38297259 10.1186/s12889-024-17663-2 PMC10832193

[62] Ickes SB, Wu M, Mandel MP, Roberts AC. Associations between social support, psychological well-being, decision making, empowerment, infant and young child feeding, and nutritional status in Ugandan children ages 0 to 24 months. Matern Child Nutr. 2018;14:e12483. 10.1111/mcn.12483 PMC6866247 28782300

[63] DeLorme AL, Gavenus ER, Salmen CR, Benard GO, Mattah B, Bukusi E, et al. Nourishing networks: A social-ecological analysis of a network intervention for improving household nutrition in Western Kenya. Soc Sci Med 1982. 2018;197:95–103. 10.1016/j.socscimed.2017.11.023 29223686

[64] Mukuria AG, Martin SL, Egondi T, Bingham A, Thuita FM. Role of social support in improving infant feeding practices in Western kenya: A Quasi-Experimental study. Glob Health Sci Pract. 2016;4:55–72. 27016544 10.9745/GHSP-D-15-00197 PMC4807749

[65] Ministry of Health (MoH). The first 1000 Days: Ensuring good health of mother and baby [Internet]. [cited 2024 May 13]. Available from: https://www.nutritionhealth.or.ke/wp-content/uploads/Downloads/The%201000%20Days%20Booklet.pdf

[66] Ministry of Health (MoH). National Social and Behaviour Change Communication: Strategy for Maternal Infant and Young Child Nutriition 2017–2020. [Internet]. Available from: https://centreforbcc.com/wp-content/uploads/2021/05/National-SBCC-Strategy-for-Maternal-and-Infant-Young-Child-Nutrition-Report-book.pdf

[67] Kihagi GW, Hansen L-S, Agure E, Muok EMO, Mank I, Danquah I, et al. Counselling is not just providing information’: perceptions of caregivers and stakeholders on the design of nutrition and health counselling interventions for families with young children in rural Kenya. BMC Health Serv Res. 2024;24:597. 38715044 10.1186/s12913-024-10872-w PMC11077832

[68] Kiprono PJ, Wario HT, Amoussa Hounkpatin W, Kaufmann BA. Food environment lens: exploring mothers’ perspectives and the dietary landscape of (Agro)-Pastoral children in kenya’s drylands. Ecol Food Nutr. 2025;64;3:122-148 10.1080/03670244.2025.2484536 40202032

[69] Matiri E, Ramirez L, Elmi A, Gaithuma J, Kavithe R, Waweru M et al. Tailoring Trials of Improved Practices (TIPs) to Improve Child Feeding and Use of Indigenous Preserved Foods in Drought-Affected Kenya: Considerations for Climate Shocks. Matern Child Nutr. n/a:e70018. 10.1111/mcn.70018 PMC12150119 40079419

[70] Nichols CE. Spaces for women: rethinking behavior change communication in the context of women’s groups and nutrition-sensitive agriculture. Soc Sci Med 1982. 2021;285:114282. 10.1016/j.socscimed.2021.114282 PMC8434409 34375897

[71] Harris-Fry HA, Paudel P, Harrisson T, Shrestha N, Jha S, Beard BJ, et al. Participatory women’s groups with cash transfers can increase dietary diversity and micronutrient adequacy during Pregnancy, whereas women’s groups with food transfers can increase equity in intrahousehold energy allocation. J Nutr. 2018;148:1472–83. 30053188 10.1093/jn/nxy109 PMC6118166

